# Autoantibody production in pregnancy: relationship with mRNA BNT162b2 immunization, active COVID-19, and pre-eclampsia

**DOI:** 10.3389/fimmu.2025.1613088

**Published:** 2025-09-25

**Authors:** Mauro César da Silva, George Tadeu Nunes Diniz, Maria Júlia da Silva Correia, Neila Caroline Henrique da Silva, Camila Rodrigues de Melo Barbosa, Ana Laura Carneiro Gomes Ferreira, Maria Inês Bezerra de Melo, Jurandy Júnior Ferraz de Magalhães, Eduardo Antônio Donadi, Ariani Impieri Souza, Norma Lucena-Silva

**Affiliations:** ^1^ Laboratory of Immunogenetics, Department of Immunology, Aggeu Magalhães Institute, Oswaldo Cruz Foundation, Recife, Brazil; ^2^ Laboratory of Computational Methods, Aggeu Magalhães Institute, Recife, Brazil; ^3^ Clinical Hospital, Federal University of Pernambuco, Recife, Brazil; ^4^ Women Health Research Group of Instituto de Medicina Integral Prof. Fernando Figueira, Recife, Brazil; ^5^ Faculdade Pernambucana de Saúde (FPS), Instituto de Medicina Integral Prof. Fernando Figueira (IMIP), Recife, Brazil; ^6^ Central Laboratory of Pernambuco, State Secretary of Health, Recife, Brazil; ^7^ Pernambuco State University, Serra Talhada, Brazil; ^8^ Clinical Immunology Division, Department of Medicine, Ribeirão Preto Medical School, University of São Paulo (USP), Ribeirão Preto, Brazil

**Keywords:** BNT162b2, pregnancy, COVID-19, autoantibodies, pre-eclampsia

## Abstract

Starting June 2021, in Brazil, the COVID-19 vaccination campaign prioritized pregnant and postpartum women to use the mRNA-based BNT162b2 (Comirnaty) vaccine, the preferred choice due to its safety profile. Although mRNA vaccines are generally safe, concerns about potential autoimmune side effects have arisen. This study aimed to assess the frequency of autoantibody production among pregnant women vaccinated with BNT162b2 compared to unvaccinated groups with active COVID-19, pre-eclampsia, and healthy control women. We studied 273 pregnant women aged 18–48 years, stratified into four groups: healthy vaccinated, healthy unvaccinated, COVID-19 positive, and pre-eclampsia. An additional control group comprised 47 healthy, non-pregnant women. Autoantibodies were detected using the HEp-2 kit (EUROIMMUN, Lübeck, SH). Statistical analysis revealed that vaccinated pregnant women exhibited a significantly lower frequency of autoantibody production compared to their unvaccinated counterparts. No significant differences in autoantibody patterns were observed between vaccinated pregnant women and the control group. Notably, control group was associated with a higher frequency of specific autoantibody patterns, including AC-4 and AC-24. These findings suggest that BNT162b2 vaccination does not increase the risk of autoimmune responses in pregnant women, contrary to some concerns. The lower frequency of autoantibody observed in vaccinated individuals may reflect beneficial immunological mechanisms, such as immune modulation and reduced viral load. Further studies are needed to explore the relationship between autoantibody production and pregnancy-related autoimmune diseases.

## 1 Introduction

In Brazil, pregnant and postpartum women became part of the priority group for immunization against COVID-19 as of June 2021. Given the restrictive use of vaccines based on adenovirus vectors, mRNA-based vaccines such as Comirnaty (Pfizer-BioNTech), also called BNT162b2, became the main vaccine used for pregnant women, which was authorized by the National Health Surveillance Agency (ANVISA) (1). The preference for BNT162b2 was due to the demonstration of the vaccine’s safety, with effective production of neutralizing antibodies against SARS-CoV-2 in the absence of reproductive toxicity in animal models, since there were no human clinical trials at that time (2). The effectiveness of the vaccine in reducing the risk of the disease and in the production of neutralizing antibodies in pregnant women has also been reported by our group, including the need for a booster to reestablish antibody levels (3).

mRNA vaccines, such as Comirnaty (Pfizer-BioNTech), use the ability of cells to translate the genetic information contained in mRNA to produce proteins. The mRNA is encapsulated in lipid nanoparticles (LNPs), and once injected into the body, the LNPs are taken up by immune cells. The mRNA is translated, and the newly synthesized spike protein is exposed on the cell surface to trigger the immune response. Synthetic mRNA is rapidly degraded in the body, not integrating into the DNA of cells and not altering the individual’s genetic material. Unlike live-attenuated vaccines, mRNA vaccines do not contain infectious viral particles, but instead deliver genetic instructions for antigen expression. This feature eliminates the risk of vaccine-derived infection and makes them particularly suitable for vulnerable populations, such as pregnant women and immunocompromised individuals (4, 5).

Despite the well-known benefits, mRNA vaccines are engineered products with a potential risk for developing autoimmune side effects, due to the possible production of autoantibodies by B cells sensitized by vaccine molecules. Although these effects may occur in any population group, pregnant women and particularly those with pre-eclampsia (PE) may present a breakdown of natural immunological tolerance due to the presence of the semi-allogeneic fetus, which may further contribute to autoimmune responses. As many viral infections, SARS-CoV-2 infection may induce autoantibodies (6–8). Because little attention has been devoted to the production of autoantibodies in pregnancy and pregnancy complications in patients exhibiting or not COVID-19, we designed a study to evaluate autoantibody production against nuclear antigens in a group of pregnant women immunized with the Comirnaty vaccine BNT162b2 (Pfizer-BioNTech) compared to unvaccinated pregnant women with active COVID-19, pre-eclampsia patients without COVID-19, and healthy control pregnant women.

## 2 Materials and methods

### 2.1 Study population and ethical considerations

**Figure 1:**
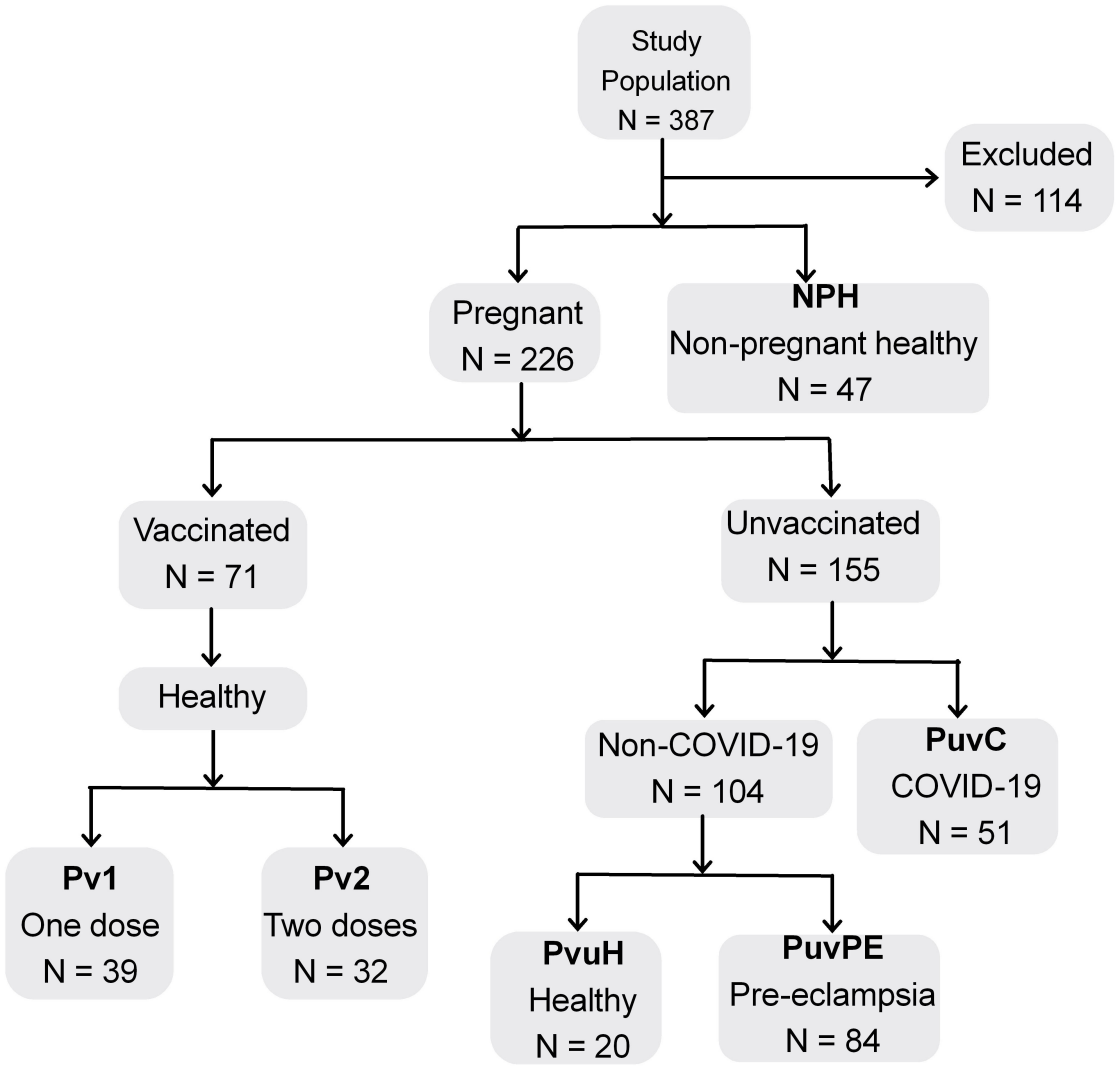
Diagram of study population and groups for comparative analysis. N, total population; Excluded, patients with comorbidities; Pv1, pregnant, 1 dose of BNT162b2; Pv2, pregnant, 2 doses of BNT162b2; PuvH, pregnant, unvaccinated, healthy (COVID-19-); PuvC, pregnant, unvaccinated, COVID-19+; PuvPE, pregnant, unvaccinated, pre-eclampsia (COVID-19-); NPH, non-pregnant, healthy with no history of COVID-19 or contact with SARS-CoV-2.

We recruited 387 participants and excluded 114 patients who tested positive for HIV or had diabetes, lupus, arthritis, or asthma that could act as confounding factors. Pre-eclampsia was the only comorbidity accepted for comparison purposes. The study population consisted of 273 women aged 18 to 48 years, with a median age of 27 years, and stratified into four major groups (**Figure 1**).

The first group included 91 healthy pregnant women, without symptoms suggestive of influenza-like illness or COVID-19 and without clinical symptoms of pre-eclampsia. They were recruited during routine prenatal consultations held at the Instituto de Medicina Integral Professor Fernando Figueira (IMIP) in Recife, Pernambuco, between August and October 2021. This group was subdivided based on the use of the BNT162b2 vaccine and the number of doses received: Pv1 encompassed 39 pregnant women vaccinated with one dose of BNT162b2 (10% in the first gestational trimester, 54% in the second, 36% in the third; median interval between last dose and sample collection: 7 weeks, range 1–26); Pv2 included 32 pregnant women vaccinated with two doses of BNT162b2 (3% first, 25% second, 72% third gestational trimester; median interval between last dose and sample collection: 4 weeks, range 1–13). The unvaccinated subgroup (PuvH) comprised 20 healthy pregnant women (20% first, 45% second, 35% third gestational trimester), recruited at the beginning of the pandemic. All 91 patients had negative RT-PCR and IgA tests for COVID-19.

The second group (PuvC) consisted of 51 pregnant women, unvaccinated, with active COVID-19 and without pre-eclampsia (0% in the first trimester, 16% in the second, 84% in the third trimester), who were invited to participate in the study upon admission to the IMIP Hospital, a reference center for cases of COVID-19 in pregnancy during the pandemic.

The third group (PuvPE) consisted of 84 pregnant women with pre-eclampsia, without COVID-19 and unvaccinated (100% of these patients were in the third trimester), recruited at the beginning of the pandemic, between April and September 2020, followed-up at the Hospital das Clínicas of the Federal University of Pernambuco in Recife.

Gestational age at the time of sample collection was described for all groups of pregnant women, while information on the interval between the last vaccine dose and sample collection was provided only for the vaccinated groups. These data can be better visualized in **Supplementary Table 1**.

The fourth group (NPH) consisted of 47 healthy, non-pregnant, unvaccinated women with no history of COVID-19 or contact with SARS-CoV-2, serving as a control group for autoantibodies in the general population. These participants were part of a study conducted between April 2016 and October 2018 at the IMIP Hospital and at the Centro Universitário Integrado de Saúde Amaury de Medeiros (CISAM Health Center).

All participants received a detailed explanation of the study and, after agreeing to participate, signed an informed consent form, completed a clinical-epidemiological questionnaire, and underwent peripheral blood collection in a tube containing EDTA anticoagulant. The sample was immediately centrifuged at 340 × g for 30 minutes, and the plasma collected and stored in a freezer at -20 °C until testing. This study was approved by the Ethics Committee of the Instituto de Medicina Integral Prof. Fernando Figueira under CAAE: 32359320.3.3001.5201, the Ethics Committee of Hospital das Clínicas of UFPE under CAAE: 32359320.3.3002.8807, and the Ethics Committee of Instituto Aggeu Magalhães under CAAE: 51111115.9.0000.5190.

### 2.2 Indirect immunofluorescence assay on HEp-2 cells

The presence of plasma antibodies against anti-nuclear antigens (ANA patterns) was evaluated using the indirect immunofluorescence HEp-2 kit (EUROIMMUN, Lübeck, SH), following the manufacturer’s instructions. This kit detects human IgG-class antibodies against nuclear, cytoplasmic, and mitotic cell components, with a screening titration of 1:100. We further performed antibody titration on some samples from patients with the most frequent positive patterns.

The results were interpreted using a Leica DMi8 fluorescence microscope (Leica Microsystems, Germany) with a FITC filter (excitation filter: 488 nm). At least three fields were analyzed for each sample, assessing the nucleus (in interphase and mitotic cells) and the cytoplasm. The pattern classification was performed by two observers according to the International Consensus on Antinuclear Antibody (ANA) Patterns (ICAP) – www.anapatterns.org/. The identified patterns were classified according to ICAP codes, based on cellular morphology and fluorescence localization: AC-0 was classified as ANA-negative; AC-1 to AC-14 and AC-29 as nuclear patterns; AC-15 to AC-23 as cytoplasmic patterns; and AC-24 to AC-28 as mitotic patterns.

### 2.3 Statistical analysis

The data in this study are presented through tables and graphs. Event occurrence probabilities were assessed using a logistic regression model with a univariate analysis approach. The magnitude of these associations was estimated using the Odds Ratio (OR) with corresponding 95% confidence intervals. All conclusions were drawn at a 5% significance level. In some comparisons, an adjustment was made considering the gestational age of the patient, and these values are represented as *Pc* (corrected p-value). The software R Core Team (2023), _R: A Language and Environment for Statistical Computing_, R Foundation for Statistical Computing, Vienna, Austria. <https://www.R-project.org/> was used for data analysis.

## 3 Results

### 3.1 Detection of autoantibodies

We initially compared the prevalence of autoantibodies in pregnant and nonpregnant women who were previously healthy, unvaccinated, and naive to SARS-CoV-2. The proportion of autoantibodies were similar in the pregnant and nonpregnant groups (PuvH vs. NPH, P = 0.8063) (**Table 1**).

**Table 1:** Frequency of positive and negative antinuclear patterns among patients.

Comparison groups	ANA negative		ANA positive		*P*	OR (CI-95%)	*Pc*	OR (CI-95%)
	N = 89	%	N = 184	%				
Influence of BNT162b2 in the group of pregnant women, in relation to NPH								
NPH	6	66.67	41	70.69	0.8063	0.83 (0.19-4.28)	n/a	n/a
PuvH	3	33.33	17	29.31				
Pv1 and Pv2	30	83.33	41	50.00	**0.0013**	5.00 (1.99-14.47)	n/a	n/a
NPH	6	16.67	41	50.00				
Pv1	14	70.00	25	37.88	**0.0147**	3.83 (1.35-12.02)	n/a	n/a
NPH	6	30.00	41	62.12				
Pv2	16	72.73	16	28.07	**0.0006**	6.47 (2.32-20.33)	n/a	n/a
NPH	6	27.27	41	71.93				
Influence of BNT162b2 on the frequency of autoantibody positivity in pregnant women								
Pv1 and Pv2	30	90.91	41	70.69	**0.0340**	4.15 (1.25-18.91)	0.0206	5.01 (1.44-23.84)
PuvH	3	9.09	17	29.31				
Pv1	14	82.35	25	59.52	0.1037	3.17 (0.87-15.29)	0.1099	3.18 (0.85-15.66)
PuvH	3	17.65	17	40.48				
Pv2	16	84.21	16	48.48	**0.0159**	5.67 (1.53-27.78)	0.0042	34.41 (4.52-809.53)
PuvH	3	15.79	17	51.52				
Pv1	14	46.67	25	60.98	0.2331	0.56 (0.21-1.45)	0.0530	0.32 (0.09-0.97)
Pv2	16	53.33	16	39.02				
Difference between healthy vaccinated pregnant women and unvaccinated pregnant women with pre-eclampsia or COVID-19.								
Pv1 and Pv2	30	50.85	41	42.71	0.3240	1.39 (0.72-2.67)	0.8228	0.91 (0.39-2.05)
PuvPE	29	49.15	55	57.29				
Pv1 and Pv2	30	58.82	41	57.75	0.9053	1.05 (0.50-2.18)	0.7587	0.88 (0.40-1.94)
PuvC	21	41.18	30	42.25				
Difference between healthy unvaccinated pregnant women and unvaccinated pregnant women with pre-eclampsia or COVID-19.								
PuvC	21	87.50	30	63.83	**0.0451**	3.97 (1.15-18.54)	n/a	n/a
PuvH	3	12.50	17	36.17				
PuvPE	29	90.62	55	76.39				
PuvH	3	9.38	17	23.61	0.1008	2.99 (0.91-13.55)	n/a	n/a
Difference between NPH and groups of pregnant women with COVID-19 or pre-eclampsia								
PuvC	21	77.78	30	42.25	**0.0027**	4.78 (1.81-14.36)	n/a	n/a
NPH	6	22.22	41	57.75				
PuvPE	29	82.86	55	57.29	**0.0094**	3.60 (1.45-10.33)	n/a	n/a
NPH	6	17.14	41	42.71				
Difference between autoantibody production during pregnancy due to pre-eclampsia and COVID-19								
PuvC	21	42.00	30	35.29	0.4382	1.33 (0.65-2.72)	0.4184	1.37 (0.64-2.90)
PuvPE	29	58.00	55	64.71				

N, number of patients; %, percentage of patients; *P*, p-value; *Pc*, corrected p-value (Gestational age–adjusted result); n/a, not applicable; OR, Odds ratio; CI, confidence interval; Pv1, pregnant, 1 dose of BNT162b2; Pv2, pregnant, 2 doses of BNT162b2; PuvH, pregnant, unvaccinated, healthy (COVID-19-); PuvC, pregnant, unvaccinated, COVID-19+; PuvPE, pregnant, unvaccinated, pre-eclampsia (COVID-19-); NPH, non-pregnant, healthy with no history of COVID-19 or contact with SARS-CoV-2.

Bold values indicate statistically significant P-values (P ≤ 0.05).

We then compared the frequency of autoantibodies in healthy nonpregnant women (NPH) with that observed in vaccinated pregnant women (Pv1 and Pv2) and found a lower frequency of autoantibody production in vaccinated pregnant women (P = 0.0013). Furthermore, when we stratified by number of BNT162b2 vaccine doses, we observed an increase in the strength of the association in the two-dose group (Pv2, P = 0.0006) with a small increase in the confidence interval, due to the decrease in cases in each comparison group. We also compared the group of vaccinated pregnant women (Pv1 and Pv2) with respect to healthy unvaccinated pregnant women (PuvH) (P = 0.0340) and the results resembled those observed with healthy, unvaccinated, non-pregnant women (NPH) (P = 0.0013) (**Table 1**).

We also observed that the frequency of autoantibodies in pregnant women with active COVID-19 (PuvC) did not differ from that of healthy vaccinated pregnant women (Pv1 and Pv2) but was lower than that of healthy unvaccinated pregnant women (PuvH, P = 0.0451) (**Table 1**).

We also investigated autoantibody production in pre-eclampsia, given the autoimmune characteristics of the disease and the impact of the disease on pregnancy success. The proportion of autoantibodies in pregnant women with pre-eclampsia was equivalent to the group of women with active COVID-19 (PuvPE vs PuvC, P = 0.4382) and lower than that of healthy, nonpregnant, unvaccinated women who had never had contact with SARS-CoV-2 (PuvPE vs NPH, P = 0.0094) (**Table 1**).

Given the limited sample size for detailed stratification within each study group, we performed an additional analysis pooling all pregnant participants and stratifying solely by gestational trimester, which revealed no significant differences in autoantibody production across the three trimesters (**Supplementary Table 2**).

These findings showed that the proportion of pregnant women who produce autoantibodies resembled that of the general population, but a lower proportion of pregnant women who have been vaccinated, had active COVID-19 or pre-eclampsia produced autoantibodies. To clarify a possible causal connection between these conditions, we analyzed the cellular pattern of the antigen-antibody reaction of the assays.

### 3.2 Cellular pattern of autoantibody labeling

**Figure 2:**
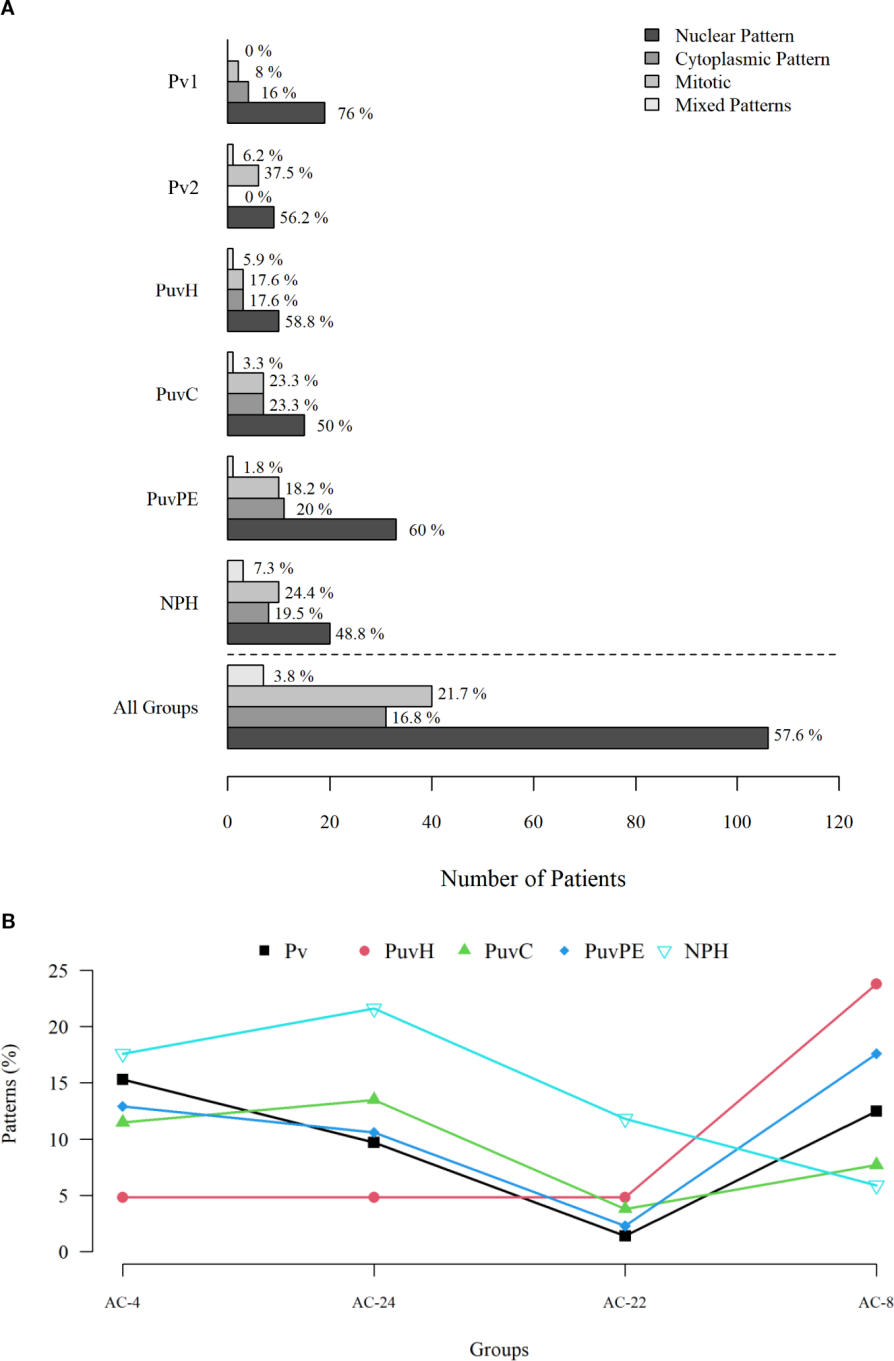
Labeling patterns of antinuclear antibodies and frequency of the main autoantibodies observed among the evaluated groups. **(A)** Graphical representation of the most frequent cellular staining patterns in our population (nuclear, cytoplasmic, mitotic, and mixed), showing their distribution across the stratified groups; no significant differences in fluorescence patterns were observed among the evaluated groups. **(B)** Most frequent positive patterns in our population (frequency ≥ 10% in at least one group). AC-4 and AC-24 were more prevalent in healthy non-pregnant women (NPH), whereas AC-8 was more frequent in unvaccinated healthy pregnant women (PuvH). The AC-22 pattern was observed at higher frequency exclusively in the NPH group. Pv, pregnant women vaccinated with 1 or 2 dose of BNT162b2; PuvH, pregnant, unvaccinated, healthy (COVID-19-); PuvC, pregnant, unvaccinated, COVID-19+; PuvPE, pregnant, unvaccinated, pre-eclampsia (COVID-19-); NPH, non-pregnant, healthy with no history of COVID-19 or contact with SARS-CoV-2.

We observed no significant differences in cellular autoantibody staining patterns between the study groups. Nuclear staining patterns predominate in positive cases in all groups (**Figure 2A**).

To better evaluate the labeling patterns, we tabulated the patterns that reached 10% frequency in at least one group and compared the frequency in the other groups (**Supplementary Table 3**). The group of healthy, non-pregnant, unvaccinated women (NPH) presented high frequencies of the AC-4 and AC-24 patterns and low frequency for the AC-8 pattern, while the group of unvaccinated pregnant women (PuvH) presented a diametrically opposite pattern. All other groups presented intermediate frequencies. The AC-4 and AC-24 frequencies were apparently more homogeneous among vaccinated pregnant women (Pv1 and Pv2), those with COVID-19 (PuvC) and pre-eclampsia (PuvPE).

Intriguing was the AC-8 pattern that appeared in 24% of healthy, unvaccinated pregnant women (PuvH), but to a lesser extent in pre-eclampsia (PuvPE), much less in vaccinated pregnant women (Pv1 and Pv2), and in those with COVID-19 (PuvC). In addition, the AC-22 pattern was found in more than 10% of the group of healthy, non-pregnant, unvaccinated women (NPH), and in less than 5% in other groups (**Figure 2B**).

### 3.3 Autoantibody titration

After observing the distribution of the most frequent patterns and considering that they could be potentially associated with the categorizing variable of each group (pregnancy, vaccination, COVID-19, and pre-eclampsia), we decided to perform titration to assess the intensity of the autoimmune response among the groups positive for the AC-4, AC-8, and AC-24 patterns (**Figure 2B**). Up to three positive samples per pattern from each comparative group were randomly selected. The maximum titration was 1:640, following the standard pre-established in Brazil according to the international consensus (9). The inclusion of additional samples and other positivity patterns was limited due to budgetary constraints, particularly related to the acquisition of reagents.

Among the 13 samples tested for each antibody positivity pattern, 23% of the AC-4 and AC-24 patterns showed positivity at titers greater than 1:100 and only 7.7% (1 sample) for the AC-8 pattern. The highest frequency of positivity for AC-4 and AC-24 was observed in the group of healthy, non-pregnant, unvaccinated women (NPH) (**Figure 2B**). High titer of autoantibody associated with the AC-4 pattern was detected in two healthy non-pregnant women (NPH), as well as in one pregnant woman with severe COVID-19 (PuvC). High titer of autoantibody associated with AC-24 was observed in one vaccinated pregnant women (Pv1 and Pv2) and in one pregnant woman with severe COVID-19 (PuvC). Additionally, high titer of autoantibody associated with AC-8 pattern was observed in one healthy, unvaccinated pregnant woman (PuvH) (**Table 2**).

**Table 2:** Indirect immunofluorescence assay on HEp-2 Cells, including titers for the most frequent patterns.

Patterns	1:100	1:320	1:640	Positive result	Negative result
	N	N	N	N (%)	N (%)
AC-4	13	3	3	19 (49%)	20 (51%)
AC-8	13	1	1	15 (38%)	24 (62%)
AC-24	13	3	2	18 (46%)	21 (54%)

Evaluation of autoimmune response intensity in patients positive for AC-4, AC-8, and AC-24 patterns, assessed through autoantibody titration, considering the high prevalence of these patterns in our study population. N, number of positive or negative patients; %, percentage of patients; IIFT, HEp-2 titers = 1:100-320-640.

## 4 Discussion

Analysis of autoantibodies in Hep-2 cells (ANA Hep-2) is currently one of the main screening methods to aid in the diagnosis of systemic rheumatic autoimmune diseases. Immortalized human laryngeal squamous cell carcinoma cells allow the detection of a wide variety of antigens, with different patterns that may be present in both autoimmune diseases and healthy individuals. The presence of antibodies against anti-nuclear antigens only configures disease when accompanied by characteristic symptoms and require additional tests to reach a specific diagnosis. The evaluations carried out in this study intended only to observe the frequency of these autoantibodies in pregnant women, in association with pregnancy, pregnancy disorders, the SARS-CoV-2 infections and particularly with the use of the mRNA-based immunogen BNT162b2 in pregnancy (9–11).

During the COVID-19 health emergency, vaccines prevented 90% of hospitalizations and deaths and 40-65% of symptomatic diseases. Although many studies demonstrate that the mRNA-based BNT162b2 vaccine has an immunogenicity profile with acceptable safety, some studies have described clinical manifestations of autoimmune diseases after immunization with this vaccine, potentially due to molecular mimicry between the vaccine, its adjuvants and the encoded autoantigens (12–16).

Autoimmune diseases are a heterogeneous set of disorders characterized by dysregulation of the immune system due to loss of self-tolerance (17). One of the mechanisms that can trigger an immune disorder capable of evolving into an autoimmune disease is mRNA-based vaccines in predisposed individuals, due to the state of chronic inflammation promoted by the constant presence of specific autoantibodies, complement activation products, platelet factor 4, polyethylene glycols, among others. In predisposed individuals, mRNA-based vaccines can induce alterations in the immune system, promoting a state of chronic inflammation due to the persistence of specific autoantibodies, complement activation products, platelet factor 4, polyethylene glycols, and other circulating molecules. Additionally, nucleic acid-based immunizers can induce autoimmune diseases due to their action as agonists of toll-like receptors 7/8/9 and by stimulating innate immunity. Notwithstanding, the exact pathogenetic mechanism that links vaccination to autoimmune diseases has not been completely understood and has been difficult to assess (12, 17–20).

The present study was designed to be discussed under two major points of views: i) evaluation of the production of autoantibodies in pregnant women immunized with the mRNA-based BNT162b2 (Pv1 & Pv2), using several control groups, including healthy unvaccinated pregnant women (PuvH), unvaccinated pregnant women with COVID-19 (PuvC), unvaccinated pregnant women with pre-eclampsia (PuvPE), and healthy, non-pregnant and unvaccinated women (NPH), and ii) the evaluation the autoantibody production during pregnancy and its relationship with the mRNA-based BNT162b2 vaccine, the presence of an active COVID-19, and the presence of pre-eclampsia.

Evidence suggests that PE shares immunological characteristics with autoimmune disorders. Autoantibodies against the angiotensin II type 1 receptor (AT1-AA) have been reported as AT1R agonists, promoting local and systemic vasoconstriction and contributing to uteroplacental hypoxia in PE. Autoimmune diseases, such as systemic lupus erythematosus (SLE) or antiphospholipid syndrome, increase the risk of developing early-onset PE (21–23). However, PE and autoimmune diseases have distinct immunological mechanisms. The soluble form of HLA-G, for example, is decreased in PE but increased in SLE, although both conditions present increased immune system reactivity (24, 25). Additionally, elevated levels of complement components and activation products, including C1q, C3a, C5a, and the terminal complex C5b-9, have been demonstrated in PE. Specific fragments, such as Bb from the alternative pathway, have been proposed as potential biomarkers for the development of the syndrome (26).

Regarding the interaction between COVID-19 and PE, SARS-CoV-2 infection may exacerbate endothelial dysfunction and systemic inflammation, central mechanisms in the pathophysiology of PE, both through impaired placentation and direct or indirect endothelial injury. In some cases, pregnant women with COVID-19 and severe pneumonia developed a PE-like syndrome, distinguishable from classic PE by the ratio of soluble fms-like tyrosine kinase 1 (sFlt-1) to placental growth factor (PlGF), a reliable marker of placental dysfunction and PE risk (27, 28).

The immune response and levels of autoantibody production following BNT162b2 vaccination have been reported to vary among individuals, with the potential to shift ANA status from positive to negative and vice versa (29). We observed that the BNT162b2 vaccine did not induce an increase in the number of pregnant women producing autoantibodies in our population. In fact, the vaccine was associated with a lower number of pregnant women producing autoantibodies. Indeed, unvaccinated healthy pregnant (PuvH) and non-pregnant (NPH) women were four to five times more likely to have positive autoantibody patterns compared to vaccinated patients (Pv1 and Pv2). We also observed fewer pregnant women with active COVID-19 (PuvC) producing autoantibodies compared to unvaccinated pregnant women.

The lower frequency of autoantibody positivity in the vaccinated population can be explained by some immunological mechanisms: i) the effect of immune modulation by regulatory T cells (Tregs), which can be stimulated by vaccines to promote immune tolerance and suppress exacerbated autoimmune responses (30), ii) the concept of trained immunity, in which the innate immune system acquires a functional memory that results in a more balanced and less inflammatory response, reducing the risk of autoantibody formation (31), iii) vaccines can reduce natural infections that trigger molecular mimicry, preventing autoimmune responses (17), iv) there is also the possibility that the targeted response of mRNA vaccines may decrease the accidental activation of autoreactive B cells, which are responsible for the production of autoantibodies, and finally v) the indirect anti-inflammatory effect of vaccines, which reduces viral load and systemic inflammation, may help decrease the activation of autoreactive B cells (32).

There is a concern about the use of vaccines in populations with autoimmune diseases, due to the possibility of worsening or progression of the pre-existing disease. However, in most cases, the benefits of the vaccine outweigh the risks (17). In the present study, we included a group of pregnant women with pre-eclampsia, due to the autoimmune nature of the disease. In general, autoantibody production was low (1:100 dilution) and present in approximately 85% of healthy pregnant and non-pregnant women, with a higher frequency of ANA positivity AC-4 and AC-24 patterns in non-pregnant women and AC-8 in pregnant women. The reduced frequency of positivity for autoantibodies associated with AC-4 (nuclear pattern) and AC-24 (mitotic pattern) in healthy pregnant women may be related to decreased autoantibody production required for a successful pregnancy. Intriguingly, among the pregnant women with higher AC-24 titers, one had received two doses of the vaccine and another had active disease. This observation should be interpreted with caution, as one of the limitations of the present study is the small number of samples with autoantibody titration, due to limited resources. In addition, we excluded patients with comorbidities such as obesity, diabetes, hypertension, lupus, and other diseases associated with autoantibody production, except for those with pre-eclampsia, because they present clinical manifestations similar to COVID-19, constituting a specific study group (33, 34).

In a study of over 7 million people, approximately 20% of the population of South Korea, Ju et al. assessed the risk of autoimmune connective tissue diseases after mRNA-based COVID-19 vaccination and found an increased risk of developing myocarditis, pericarditis, and thrombocytopenia in the vaccinated population when compared with the control population, but there were no differences in the development of autoimmune diseases. Thus, they suggested that the mRNA-based COVID-19 vaccine is not significantly associated with the development of autoimmune diseases involving connective tissue. Vaccination may, in fact, represent an environmental factor capable of triggering autoimmune diseases, but only in individuals with genetic susceptibility, and not in healthy individuals (12). Therefore, it is essential that future studies include the evaluation of genetic susceptibility to immune autoreactivity through immunogenetic approaches, such as the analysis of specific HLA alleles (for example, HLA-DRB1*15, previously associated with an increased risk of autoimmune diseases), as well as variants in genes involved in the regulation of immune responses. Although these investigations are beyond the scope of our study, they can provide explanations for the presence of autoantibodies in groups considered healthy and help identify individuals genetically predisposed to developing autoimmune responses potentially triggered by factors such as SARS-CoV-2 infection, pre-eclampsia, or mRNA-based vaccination platforms (35, 36).

Our study presents a methodological limitation, as it relies exclusively on the indirect immunofluorescence assay on HEp-2 cells (IIF-HEp-2), which, although recognized for its high sensitivity in screening for antinuclear autoantibodies (37), does not allow the identification of autoantibodies directed against functionally relevant soluble antigens, such as cytokines (e.g., IFN-α, IL-6) or regulators of the antiviral immune response (38, 39). Nevertheless, the cellular staining patterns observed in the HEp-2 assay follow internationally accepted classification criteria and continue to be widely used in both clinical and research contexts, including as predictors of risk for systemic autoimmune diseases. Thus, the findings described here provide important preliminary data on the frequency and types of autoreactivity in pregnant women under different immunological conditions, serving as a basis for future investigations using complementary methodologies.

We concluded that the use of the mRNA-based BNT162b2 vaccine in our pregnant population was not associated with an increased frequency of autoantibody production. However, it is important to note that we were unable to assess whether similar results would be observed with other mRNA vaccines, such as Moderna’s mRNA-1273, since ANVISA restricted the use of COVID-19 vaccines in Brazilian pregnant women to BNT162b2 during the study period for safety reasons (1). This comparison remains an important and open question. To date, we have not identified studies that directly compare the impact of different mRNA vaccines on autoantibody production during pregnancy. Although BNT162b2 and mRNA-1273 share the same mRNA-based platform and immunological principles, differences in formulation, mRNA dose (30 µg vs. 100 µg), and dosing intervals may modulate the magnitude and duration of the immune response (including potential effects on immune tolerance) which should be considered in future studies (40, 41).

Misconceptions about the vaccine-mediated immunization process reduce public confidence and, consequently, adherence to vaccination programs (17). Some unanswered questions that warrant further investigation in future studies relate to the influence of gestational age and the timing of vaccine dose administration on autoantibody production, considering that fetal immune tolerance fluctuates throughout pregnancy. It is well established that the first gestational trimester is characterized by a pro-inflammatory immune profile (necessary for embryo implantation), the second trimester shifts to a predominantly anti-inflammatory state (to support fetal development), and the third gestational trimester returns to a pro-inflammatory profile (to prepare for labor) (42). The frequency of positivity for autoantibody patterns produced in the three trimesters of pregnancy in our study population was similar. However, the role of autoantibodies associated with the AC-8 pattern in both physiological and pathological pregnancy remains unclear. This staining pattern indicates nucleolar binding (a region involved in ribosomal RNA synthesis), which raises the hypothesis of a potential interaction with mRNA-based vaccine activity.

We consider that the findings presented here provide relevant preliminary evidence that vaccination with BNT162b2 is not associated with increased immunological autoreactivity in pregnant women. This may contribute to strengthening public and healthcare professionals’ confidence in maternal immunization. As demonstrated, our results do not address all the existing gaps regarding mRNA vaccination and autoimmunity during pregnancy. However, they represent an important starting point for future investigations aiming to further explore the underlying immunological mechanisms, potential genetic susceptibility factors, and the unique features of the maternal immune response. Given the relevance of this topic to maternal-fetal health and vaccine safety, additional studies are essential to clarify risks, validate findings, and inform evidence-based clinical practices.

## Data Availability

The original contributions presented in the study are included in the article/**Supplementary Material**. Further inquiries can be directed to the corresponding author.
