# Supplementary material for: Autoantibody production in pregnancy: relationship with mRNA BNT162b2 immunization, active COVID-19, and pre-eclampsia
Source: Front Immunol. 2025 Sep 25;16:1613088. doi: 10.3389/fimmu.2025.1613088 (PMC12507805; doi:10.3389/fimmu.2025.1613088)
Supplement: Supplementary file 1 [file Table1.docx]

Supplementary Material

# Supplementary Tables

**Suplementary Table 1** – Gestational age at the time of sample collection and interval between vaccination and collection among vaccinated pregnant women

**Legend:** N = number of patients; % = percentage of patients; Pv1 = pregnant, 1 dose of BNT162b2; Pv2 = pregnant, 2 doses of BNT162b2; PuvH = pregnant, unvaccinated, healthy (COVID-19-); PuvC = pregnant, unvaccinated, COVID-19+; PuvPE = pregnant, unvaccinated, pre-eclampsia (COVID-19-); NPH = non-pregnant, healthy with no history of COVID-19 or contact with SARS-CoV-2.

| Gestational age | Pv1 | Pv2 | PuvH | PuvC | PuvPE |
| --- | --- | --- | --- | --- | --- |
|  | N (%) | N (%) | N (%) | N (%) | N (%) |
| 3rd Trimester (28-41 weeks) | 14 (36%) | 23 (72%) | 7 (35%) | 43 (84%) | 84 (100%) |
| 2nd Trimester (13 to 27 weeks) | 21 (54%) | 8 (25%) | 9 (45%) | 8 (16%) | - |
| 1st trimester (<13 weeks) | 4 (10%) | 1 (3%) | 4 (20%) | - | - |
| Interval between last dose and collection (weeks) | (1 - 26) | (1 - 13) | - | - | - |
| Median time between last dose and collection (weeks) | 7 | 4 | - | - | - |

**Suplementary Table 2** – Frequency of positive and negative antinuclear antibody patterns according to gestational age

**Legend:** N = number of patients; % = percentage of patients; *P* = p-value; OR = Odds ratio; CI = confidence interval; Ref. = Reference.

|  |  |  |  |  | | |  |  | |  | |  | |  |  | |  |
| --- | --- | --- | --- | --- | --- | --- | --- | --- | --- | --- | --- | --- | --- | --- | --- | --- | --- |
| **Gestacional Age** | | | | | **ANA Negative** | | | | **ANA Positive** | | | | ***P*** | | | **OR (CI-95%)** | |
|  |  |  |  |  | **N** | **%** | | | **N** | | **%** | |  |  |  |  |  |
| **Trimester** | | | | |  |  | | |  | |  | |  | | |  | |
| 1st | | | | | 3 | 3.61 | | | 6 | | 4.20 | | Ref. | | |  | |
| 2nd | | | | | 21 | 25.30 | | | 25 | | 17.48 | | 0.4985 | | | 0.60 (0.11-2.55) | |
| 3rd | | | | | 59 | 71.08 | | | 112 | | 78.32 | | 0.9426 | | | 0.95 (0.19-3.73) | |

**Suplementary Table 3** – Most frequent patterns among comparison groups

**Legend:** N = number of patients; % = percentage of patients; AC-0 = negative; Pv1 = pregnant, 1 dose of BNT162b2; Pv2 = pregnant, 2 doses of BNT162b2; PuvH = pregnant, unvaccinated, healthy (COVID-19-); PuvC = pregnant, unvaccinated, COVID-19+; PuvPE = pregnant, unvaccinated, pre-eclampsia (COVID-19-); NPH = non-pregnant, healthy with no history of COVID-19 or contact with SARS-CoV-2.

| **Patterns** | **Pv1 & Pv2** | | **PuvH** | | **PuvC** | | **PuvPE** | | **NPH** | |
| --- | --- | --- | --- | --- | --- | --- | --- | --- | --- | --- |
|  | **N** | **%** | **N** | **%** | **N** | **%** | **N** | **%** | **N** | **%** |
| **AC-0** | 30 | 41.7 | 3 | 14.3 | 21 | 40.4 | 29 | 34.1 | 6 | 11.8 |
| **AC-4** | 11 | 15.3 | 1 | 4.8 | 6 | 11.5 | 11 | 12.9 | 9 | 17.6 |
| **AC-24** | 7 | 9.7 | 1 | 4.8 | 7 | 13.5 | 9 | 10.6 | 11 | 21.6 |
| **AC-2** | 2 | 2.8 | 1 | 4.8 | 1 | 1.9 | 1 | 1.2 | 3 | 5.9 |
| **AC-8** | 9 | 12.5 | 5 | 23.8 | 4 | 7.7 | 15 | 17.6 | 3 | 5.9 |
| **AC-22** | 1 | 1.4 | 1 | 4.8 | 2 | 3.8 | 2 | 2.3 | 6 | 11.8 |
| **Total** | 64 | 88.9 | 14 | 66.6 | 44 | 84.6 | 71 | 83.5 | 43 | 84.3 |
